# Personality Traits Affect Teaching Performance of Attending Physicians: Results of a Multi-Center Observational Study

**DOI:** 10.1371/journal.pone.0098107

**Published:** 2014-05-20

**Authors:** Renée A. Scheepers, Kiki M. J. M. H. Lombarts, Marcel A. G. van Aken, Maas Jan Heineman, Onyebuchi A. Arah

**Affiliations:** 1 Professional Performance Research Group, Centre for Evidence-Based Education, Academic Medical Centre, University of Amsterdam, Amsterdam, the Netherlands; 2 Department of Developmental Psychology, Utrecht University, Utrecht, the Netherlands; 3 Board of Directors, Academic Medical Centre, University of Amsterdam, Amsterdam, the Netherlands; 4 Department of Epidemiology, The Fielding School of Public Health, University of California Los Angeles (UCLA), Los Angeles, California, United States of America; University of Minho, Portugal

## Abstract

**Background:**

Worldwide, attending physicians train residents to become competent providers of patient care. To assess adequate training, attending physicians are increasingly evaluated on their teaching performance. Research suggests that personality traits affect teaching performance, consistent with studied effects of personality traits on job performance and academic performance in medicine. However, up till date, research in clinical teaching practice did not use quantitative methods and did not account for specialty differences. We empirically studied the relationship of attending physicians' personality traits with their teaching performance across surgical and non-surgical specialties.

**Method:**

We conducted a survey across surgical and non-surgical specialties in eighteen medical centers in the Netherlands. Residents evaluated attending physicians' overall teaching performance, as well as the specific domains learning climate, professional attitude, communication, evaluation, and feedback, using the validated 21-item System for Evaluation of Teaching Qualities (SETQ). Attending physicians self-evaluated their personality traits on a 5-point scale using the validated 10-item Big Five Inventory (BFI), yielding the Five Factor model: extraversion, conscientiousness, neuroticism, agreeableness and openness.

**Results:**

Overall, 622 (77%) attending physicians and 549 (68%) residents participated. Extraversion positively related to overall teaching performance (regression coefficient, B: 0.05, 95% CI: 0.01 to 0.10, *P* = 0.02). Openness was negatively associated with scores on feedback for surgical specialties only (B: −0.10, 95% CI: −0.15 to −0.05, *P*<0.001) and conscientiousness was positively related to evaluation of residents for non-surgical specialties only (B: 0.13, 95% CI: 0.03 to 0.22, *p* = 0.01).

**Conclusions:**

Extraverted attending physicians were consistently evaluated as better supervisors. Surgical attending physicians who display high levels of openness were evaluated as less adequate feedback-givers. Non-surgical attending physicians who were conscientious seem to be good at evaluating residents. These insights could contribute to future work on development paths of attending physicians in medical education.

## Introduction

Residents carry out much of daily patient care, while being learners at the same time. Therefore, patient care quality benefits from adequate supervision of residents [1], [2]. To assess adequate supervision in residency training, attending physicians are increasingly evaluated on their clinical teaching performance [3]–[10]. Systems and tools for robust evaluation of teaching performance are available and high and low performing attending physicians can be identified [9], [11].

Research suggests that attending physicians who are younger, female and spend more time on teaching and conducting research, are more favorably evaluated on their teaching performance [12]. In addition, there is research indicating that high performing attending physicians could be identified by their personality traits [13], which is in line with well documented personality research in the field of job performance[14]–[19] and academic performance in medicine [20]–[24]. Yet, personality research on teaching performance of attending physicians is limited and uses qualitative methods only. Understanding the plausible link between them can shed critical light on opportunities and policies regarding the development paths and career planning of attending physicians in medical education.

What are personality traits and in what way could they affect teaching of attending physicians? Personality traits can be categorized in five comprehensive domains, called the Five Factor Model: conscientiousness, extraversion, emotional stability, agreeableness and openness [17], [25], [26]. Conscientiousness refers to dependability and includes traits such as being responsible, organized, orderly and thorough [14]. Employees who are conscientious take responsibility for their work, accomplishing their work tasks more thoroughly and orderly. Ultimately, university teachers who are orderly are better evaluated [27] and physicians who are conscientious are thought to be good teachers in medicine [13]. Higher levels of conscientiousness of attending physicians could positively influence their teaching in residency training, as training of residents is an important responsibility[13]. Yet, the role of conscientiousness in clinical teaching practice remains unexplored.

Extraversion means being sociable, talkative, outgoing and active [17]. Extraverted people perform better in professions involving social interaction [15]. Unlike research on the other four personality traits, research on the working mechanism of extraversion with respect to social interaction provided a neurobiological explanation. That is, extraverts have lower cortical activity than introverts. This makes extraverts seek to attain a higher level of arousal by increasing social activity, while the higher levels of cortical activity of introverts make them more comfortable with fewer impulses [28]–[33]. Subsequently, extraverted people can function more efficiently in the presence of others [34]. This is seen in research showing that university teachers who are extraverted are better evaluated [27], [35] and attain higher levels of teaching effectiveness [36]. There is a need for empirical work on the impact of extraversion on teaching performance of attending physicians involved in residency training.

Emotional stability involves high levels of self-esteem, positive affect and low levels of stress and anxiety. Indeed, a lack of emotional stability is associated with high levels of stress, anxiety and neuroticism [14]. Research suggests that university teachers who are emotionally unstable are hindered in their performance by their insecurities and anxieties.[35] Also, anxiety has been shown to affect working memory adversely [37]–[39] and to deplete available cognitive resources, which tend to hinder in adequate coping of stressful situations [40], [41]. Therefore, emotionally unstable people are more likely to perceive stressful situations as threats [41], [42]. In contradiction, emotionally stable people are more likely to perceive stressful situations as challenging, as they experience less negative emotions and do not deplete cognitive resources to deal with the situation [41]. In clinical teaching, attending physicians must pay attention to both patient well-being and resident training quality, and must adequately respond to arising circumstances. These are demanding for attending physicians' cognitive resources. As such, being emotionally unstable might inhibit teaching performance of attending physicians, while emotional stability could facilitate their teaching. Still, there is little empirical investigation into the impact of emotional stability on clinical teaching performance in residency training.

Another personality trait that remains unexplored in the context of clinical teaching is agreeableness. Agreeableness refers to friendliness and includes being kind, cooperative, flexible and tolerant. Research suggests that agreeableness has positive relations with work performance where social interaction is part of the job, especially when it involves helping and cooperating with others [14], [16]. This is likely to be the case in residency training. Agreeable attending physicians are thought to be good in teaching and acting on residents' personal learning needs, because of their natural tendency to take into account the interests of other people [15]. This is consistent with findings that good teachers in medicine are personable, altruistic and consider others' viewpoints [13]. Yet, no research has quantified the relationship between agreeableness and teaching performance of attending physicians.

Finally, openness is a personality trait that refers to being open and receptive to experience. Openness is associated with being imaginative, cultured, curious, and broad-minded. Findings suggesting that curiosity benefits teaching effectiveness [27] are in line with possible benefits of openness: attending physicians who are open and curious to residents' progress could be stimulating teachers. This still needs to be demonstrated.

Although personality traits characterize individuals, attending physicians do not function as individuals only — they work in teams within departments, delivering specialized patient care and medical training. The clinical specialty establishes a specific professional context, not only for the nature of patient care that varies across specialties, but also for interpersonal behaviors towards and interactions with patients [43]–[46]. In addition, teaching performance of attending physicians is differently evaluated across specialties [6]. What works for one specialty, does not necessarily work for another specialty. This is in line with Nettle's cost-benefit trade-off model, which states that costs and benefits of personality traits depend on the context in which they are expressed [47], [48]. Subsequently, a certain personality trait could be beneficial for the teaching of residents within one specialty, but could come with costs within another specialty. Still, specialty dependent effects of personality on teaching performance of attending physicians are unexplored.

Overall, since previous research suggests that personality traits could affect teaching performance in (mostly) non-clinical settings, there is a critical need for examining these in residency training. Moreover, the little existing research done in clinical teaching settings used qualitative methods only, making it nearly impossible to make inferences based on quantitative evidence. Moreover, nothing is known about differences between specialties in terms of plausible links between personality traits and teaching performance. Therefore, the objective of this study is to examine the relationship of personality traits with teaching performance of attending physicians within and across surgical and non-surgical specialties. We hypothesize that conscientiousness, extraversion, emotional stability, agreeableness, and openness all positively affect teaching performance. Since the differences between surgical and non-surgical specialties on this matter had not been documented in the literature, we had no specific expectations, electing for an explorative approach to this issue.

## Materials and Methods

### Study population

This multicenter study was conducted at 61 different residency programs, covering 7 surgical and 18 non-surgical specialties, in 2 academic and 16 non-academic medical centers in the Netherlands, from May to December 2012. We invited 815 residents and 819 attending physicians by email and mentioned the formative purpose and use of the evaluations and stressed the confidential and voluntary character of participation.

### Measures

To measure teaching performance, we conducted a survey using the well-published System for Evaluation of Teaching Qualities (SETQ), a system for continuous evaluation of attending physicians, which is used by approximately 6000 residents and attending physicians representing 45 medical centers in the Netherlands. The details of instrument development are described elsewhere, showing that the instruments provide reliable and valid evaluations of the teaching qualities of attending physicians [4], [5], [7], [8]. Residents evaluated attending physicians in five domains, using 21 items: creating a motivating learning climate, displaying professional attitudes towards residents, communicating learning goals, evaluating residents, and giving them feedback. All items could be filled out on a 5-point-scale, ranging from “Totally disagree” to “Totally agree”, with an additional option “I cannot judge”. Residents could choose which and how many attending physicians to evaluate.

Attending physicians self-reported their personality traits using the shortened version of the Big Five Inventory (BFI-10),[49] as an additional and optional questionnaire attached to SETQ. The BFI-10 measures personality in five domains according to the Five Factor Model: conscientiousness, extraversion, emotional stability, agreeableness and openness [25]. Attending physicians could self-report their personality scales on a 5-point scale.

Taking into account BFI authors' recommendations, we added an extra item for the subscale agreeableness in order to safeguard internal consistency for this subscale, as it showed less internal consistency than the other personality subscales [49]. This way, our BFI contained eleven items, instead of ten. Because the original version of the BFI-10 was not yet validated in Dutch, two researchers independently translated the English instrument forward and agreed upon one Dutch version. Two other bilingual researchers performed back-translation of that version. Based on the minor differences between the back-translation and the original English instrument, we adjusted the forward translation into the final Dutch version of our BFI-11.

Attending physicians' gender was considered as a confounding variable, as research showed gender differences in personality as well as in teaching performance [12], [50]. We created a dummy for gender, with male as the reference category. Furthermore, we used age as a confounding variable as well, because research demonstrated differences in both personality traits and teaching performance across age [51]. For attending physicians' specialty, we created two categories, namely surgical and non-surgical specialties. Surgical specialties included: plastic and reconstructive surgery, neurosurgery, general surgery, orthopedics, urology, ophthalmology, otorhinolaryngology, obstetrics and gynecology. Non-surgical specialties included: internal medicine, gastroenterology, neurology, cardiology, pulmonology, pediatrics, dermatology, psychiatry, emergency medicine, radiology, radiotherapy, anesthesiology, rehabilitation medicine, pathology, nuclear medicine and clinical genetics.

### Analytical strategies

First, we aggregated teaching performance evaluations of different residents on the level of individual attending physicians, which resulted in average scores on teaching performance items for each attending physician. We calculated means and medians for the overall as well as domain sum scores when at least two third of the items were completed. [We found no differences in multivariable analyses using means versus median scores; hence, we focus on analysis using on mean scores.] Next, we described the study sample using applicable analytical techniques. Using a random half of the sample, we then explored whether the five-domain structure of the original BFI-10 also applied to our sample data by conducting a principal components factor analysis, with promax rotation and retaining only factors with eigenvalue >1. With the second half of the sample data, we conducted confirmatory factor analysis of the factor structure that emerged from the previous step. We assessed goodness of fit using Comparative fit index (CFI), Tucker-Lewis Index (TLI), root mean square error of approximation (RMSEA), and standardized root mean square residual (SRMR). CFI >0.95, TLI >0.95, RMSEA <0.06 and SRMR <0.08 were taken to indicate good fit. We also accounted for clustering within hospitals and assessed equivalence across groups in the sample (for example, specialties).

The six scores for overall teaching performance and its five domains were used as outcomes in subsequent multivariable analysis. To quantify the impact of attending physicians' personality traits on their teaching performance as evaluated by residents, we performed multivariable adjusted regression analyses using generalized estimating equations (GEEs). Using GEE was appropriate because of their capacity to account for data nesting or clustering at different levels, such as attending physicians being part of specific departments in different hospitals. We, therefore, regressed (i) overall teaching performance on BFI-subscales, and (ii) each of the five domains of teaching performance on BFI-subscales, for all specialties combined. These two sets of models were repeated for surgical and non-surgical specialty groupings separately. To test for differences between the samples, we performed fit models with interaction terms. For this purpose, we used product terms involving each personality trait and surgical specialty (with non-surgical as reference). We controlled for gender and age by conditioning on them in the regression models. To deal with the varying number of resident evaluations of teaching performance per attending physician, we checked whether the results were sensitive to this variable, by adjusting the analyses for this. Because not all participating attending physicians filled out the questionnaires (participation was voluntary), we controlled for a possible selection bias by reweighting the analyses for selection probability. All analyses were conducted in IBM SPSS Statistics version 20 (IBM Corporation, 2011), Stata version 13.1 for Mac OS (StataCorp LP, 2013), and R version 3.0.2 (The R Foundation for Statistical Computing Platform, 2013).

### Ethics statement

The institutional ethical review board of the Academic Medical Centre of the University of Amsterdam (AMC) waived ethical approval for this study.

## Results

In total, 560 (68%) residents filled out 4305 evaluations of 805 attending physicians: 622 (77%) attending physicians participated, of whom 515 (83%) self-reported their personality traits (see Table 1). The mean number of resident evaluations per attending physician was 5.83, which means that criteria for reliable evaluations were satisfied [4], [5], [7], [8].

**Table 1:** Descriptive statistics of the study sample.

		N
**Setting**	Medical centres (academic/non-academic)	18 (2/16)
	Residency programs	61
	Surgical/non-surgical specialties	7/18
**Participants**	Residents participated (% of total invited)	560 (68%)
	Resident evaluations	4368
	Clinical faculty members participated (% of total invited)	636 (78%)
	Clinical faculty members evaluated by residents	805
	Mean resident evaluations per clinical faculty member	5.43
	Surgical/non-surgical clinical faculty members	281/385
	Female/male clinical faculty members (% females)	252/366 (41%)
	Mean clinical faculty members' age	48

According to the factor analysis, the original five-factor structure of the BFI-11 was confirmed for our specific study sample (see Table 2). The positively recoded agreeableness item (“tends to find fault with others”) had a lower factor loading than 0.70 (0.41) and was excluded from the agreeableness scale, still remaining in two items for this scale (see Table 2). Furthermore, we concluded a high degree of specificity of the subscales, as inter-scale correlations did not reach higher levels than *r* = 0.22. Results of the CFA supported the foregoing structure with acceptable goodness of fit indices: CFI  = 0.977; TLI  = 0.959; RMSEA  = 0.032; SRMR  = 0.034. There were no differences in factor structure across groups such as specialties.

**Table 2:** Means, standard deviation (SD), item factor loadings and inter-scale correlations of the SETQ and BFI (sub) scales.

	Mean	SD	Factor loadings	Inter-scale correlations						
				EX	EM		CO		AG	OP
**Personality traits** #										
*Extraversion (EX)*	3.48	0.88		1						
is reserved^a^			0.89							
is outgoing, sociable			0.75							
*Emotional stability (EM)*	3.87	0.74		0.22**	1					
is relaxed, handles stress well			0.81							
gets nervous easily^a^			0.82							
*Conscientiousness (CO)*	4.26	0.62		0.06	0.09*		1			
tends to be lazy^a^			0.80							
does a thorough job			0.76							
*Agreeableness (AG)*	3.90	0.58		0.19**	0.15**		0.15**		1	
is generally trusting			0.81							
is considerate and kind to almost everyone			0.76							
*Openness (OP)*	3.43	0.87		0.09*	0.05		−0.02		0.07	1
has few artistic interests^a^			0.83							
has an active imagination			0.82							
				TP	LC	PA		COM	EV	FB
**Teaching performance**										
*Overall teaching performance (TP)*	3.85	0.42		1						
*Learning climate (LC)*	3.90	0.47		0.883**	1					
encourages residents to participate actively in discussions										
stimulates residents to bring up problems										
motivates residents to study further										
stimulates residents to keep up with the literature										
prepares well for teaching presentations and talks										
*Professional attitude (PA)*	4.28	0.48		0.646**	0.455**	1				
listens attentively to residents										
is respectful towards residents										
is easily approachable during on-calls										
*Communication of learning goals (COM)*	3.45	0.59		0.861**	0.689**	0.417**		1		
states learning goals clearly										
states relevant goals										
prioritizes learning goals										
repeats stated learning goals periodically										
*Evaluation of residents (EV)*	3.79	0.47		0.848**	0.745**	0.364**		0.681**	1	
evaluates residents' specialty knowledge regularly										
evaluates residents' analytical abilities regularly										
evaluates residents' application of knowledge to specific patients regularly										
evaluates residents' medical skills regularly										
*Feedback (FB)*	3.94	0.49		0.870**	0.666**	0.660**		0.674**	0.657**	1
gives positive feedback to residents regularly										
gives corrective feedback to residents										
explains why residents are incorrect										
offers suggestions for improvement										

# Goodness of fit indices from confirmatory factor analysis: CFI  = 0.977; TLI  = 0.959; RMSEA  = 0.032; SRMR  = 0.034. There were no material differences across specialties.

*Correlation is significant at the 0.05 level.

**Correlation is significant at the 0.01 level.

a Negative formulated item; score was positively recoded before factor analyzing.

Attending physicians' extraversion was positively associated with their *overall teaching performance* (see Table 3). For *learning climate*, both extraversion and conscientiousness were positively related, while agreeableness was negatively related. The *professional attitude* of attending physicians was positively associated with their degree of agreeableness. Conscientiousness and extraversion were positively associated with attending physicians' scores on *communication of learning goals*. Attending physicians' extraversion positively influenced their scores on *feedback* and *evaluation of residents*. Agreeableness negatively influenced scores on evaluation of residents.

**Table 3:** Unstandardized regression coefficients (B) of associations of attending physicians' personality traits with teaching performance domains.

	All specialties		Surgical specialties		Non-surgical specialties		Personality-Specialty^c^
	B (95% CI^b^)	p	B (95% CI^b^)	p	B (95% CI^b^)	p	p
	***Overall teaching performance***						
**Extraversion**	0.05 (0.01–0.10)	0.019	0.02 (−0.04–0.08)	0.505	0.07 (0.00–0.13)	0.048	0.284
**Emotional stability**	0.00 (−0.06–0.06)	0.983	−0.02 (−0.09–0.04)	0.471	0.03 (−0.07–0.13)	0.562	0.239
**Conscientiousness**	0.05 (−0.01–0.10)	0.115	0.02 (−0.05–0.09)	0.581	0.08 (−0.02–0.17)	0.109	0.552
**Agreeableness**	−0.03 (−0.08–0.02)	0.194	0.02 (−0.05–0.09)	0.532	−0.06 (−0.13–0.00)	0.057	0.250
**Openness**	−0.03 (−0.08–0.01)	0.147	−0.08 (−0.13–0.03)	0.004	−0.01 (−0.07–0.06)	0.830	0.050
N	494		229		265		
QICC^a^	92.748		42.711		64.287		
	***Learning climate***						
**Extraversion**	0.06 (0.02–0.11)	0.009	0.03 (−0.04–0.09)	0.455	0.08 (0.02–0.15)	0.017	0.234
**Emotional stability**	0.01 (−0.06–0.08)	0.775	−0.02 (−0.10–0.07)	0.701	0.06 (−0.04–0.17)	0.246	0.114
**Conscientiousness**	0.07 (0.01–0.14)	0.032	0.04 (−0.03–0.12)	0.238	0.11 (−0.00–0.22)	0.059	0.601
**Agreeableness**	−0.05 (−0.11–0.00)	0.049	−0.01 (−0.09–0.07)	0.854	−0.08 (−0.15–0.01)	0.020	0.432
**Openness**	−0.02 (−0.07–0.04)	0.497	−0.05 (−0.12–0.03)	0.244	−0.02 (−0.09–0.06)	0.658	0.354
N	499		229		270		
QICC^a^	120.090		57.107		74.418		
	***Professional attitude***						
**Extraversion**	−0.01 (−0.06–0.04)	0.664	−0.04 (−0.10–0.02)	0.209	0.01 (−0.07–0.08)	0.849	0.176
**Emotional stability**	0.02 (0.03)	0.514	−0.01 (−0.08–0.05)	0.743	0.04 (−0.06–0.15)	0.445	0.409
**Conscientiousness**	−0.01 (−0.08–0.05)	0.688	−0.00 (−0.08–0.07)	0.901	−0.02 (−0.14–0.10)	0.773	0.897
**Agreeableness**	0.10 (0.05–0.16)	0.000	0.10 (0.04–0.16)	0.001	0.11 (0.02–0.20)	0.014	0.430
**Openness**	−0.04 (−0.07–0.00)	0.058	−0.08 (−0.12–0.04)	0.000	−0.01 (−0.07–0.05)	0.847	0.050
N	467		216		251		
QICC^a^	93.654		40.161		68.534		
	***Communication of learning goals***						
**Extraversion**	0.07 (0.02–0.13)	0.009	0.05 (−0.02–0.13)	0.183	0.08 (0.04)	0.064	0.884
**Emotional stability**	−0.04 (−0.11–0.04)	0.329	−0.03 (−0.13–0.06)	0.507	−0.03 (0.06)	0.662	0.905
**Conscientiousness**	0.11 (0.02–0.19)	0.017	0.06 (−0.05–0.16)	0.288	0.16 (0.07)	0.029	0.447
**Agreeableness**	−0.07 (−0.15–0.01)	0.073	−0.00 (−0.12–0.11)	0.982	−0.12 (0.05)	0.016	0.222
**Openness**	−0.03 (−0.09–0.04)	0.396	−0.08 (−0.16–0.01)	0.035	0.00 (0.05)	0.0947	0.122
N	487		229		258		
QICC^a^	167.866		69.606		66.910		
	***Feedback***						
**Extraversion**	0.06 (0.00–0.12)	0.037	0.02 (−0.04–0.09)	0.438	0.07 (−0.02–0.16)	0.105	0.297
**Emotional stability**	−0.00 (−0.07–0.06)	0.870	−0.05 (−0.11–0.01)	0.079	0.02 (−0.10–0.14)	0.733	0.167
**Conscientiousness**	0.03 (−0.03–0.10)	0.319	0.03 (−0.05–0.11)	0.507	0.06 (−0.06–0.17)	0.340	0.858
**Agreeableness**	−0.02 (−0.06–0.06)	0.982	0.04 (−0.03–0.11)	0.238	−0.02 (−0.11–0.07)	0.678	0.743
**Openness**	−0.04 (−0.08–0.01)	0.163	−0.10 (−0.15–0.05)	0.000	0.01 (−0.06–0.08)	0.805	0.008
N	501		229		272		
QICC^a^	123.095		47.345		88.938		
	***Evaluation of residents***						
**Extraversion**	0.06 (0.01–0.11)	0.011	0.02 (−0.05–0.09)	0.531	0.08 (0.01–0.15)	0.019	0.167
**Emotional stability**	0.02 (−0.03–0.08)	0.429	0.00 (−0.08–0.08)	0.955	0.06 (−0.04–0.15)	0.219	0.175
**Conscientiousness**	0.04 (−0.03–0.11)	0.299	−0.04 (−0.13–0.04)	0.333	0.13 (0.03–0.22)	0.014	0.039
**Agreeableness**	−0.08 (−0.14– −0.02)	0.008	0.00 (−0.08–0.08)	0.999	−0.14 (−0.21–0.06)	0.000	0.133
**Openness**	−0.04 (−0.09–0.01)	0.119	−0.10 (0.03–0.04)	0.002	−0.01 (−0.08–0.06)	0.818	0.040
N	500		229		271		
QICC^a^	127.259		54.303		85.491		

All analyses were controlled for confounders, namely, gender and age.

a QICC  =  Corrected Quasi Likelihood under Independence Model Criterion: information criterion in smaller-is-better form; that is, the model with the smallest QICC has the best goodness of fit.

b CI  =  confidence interval.

c Interaction term of personality (trait) and specialty.

We found that there were differences of personality traits' associations with teaching performance across surgical and non-surgical specialty groupings (see Table 3). When testing whether these associations across specialties differed significantly (see Table 3, under the column personality*specialty), it could be concluded that openness was negatively associated with scores on *feedback* for surgical specialties and not for surgical specialties (this is also visually presented in Figure S1). Conscientiousness was positively related to *evaluation of residents* for non-surgical specialties, while this was not the case for surgical specialties (Figure S2). In addition, openness was more negatively related to evaluation of residents for surgical attending physicians than for their surgical peers (Figure S3).

The foregoing results did not change materially after further accounting for the varying number of resident evaluations per attending physician. The findings were also robust following sensitivity analysis for selection bias.

## Discussion

### Main findings

We hypothesized that conscientiousness, extraversion, emotional stability, agreeableness and openness would positively affect teaching performance of attending physicians. In general, the results suggest that different personality traits have different – both positive and negative - effects on different aspects of teaching performance. Of all findings, the most outstanding one is that extraverted attending physicians are evaluated as better teachers, both on general and specific teaching performance. As for differences between specialties, surgeons who display higher levels of openness received lower scores on their quality of giving feedback and evaluation of residents. Non-surgical attending physicians who are more conscientious appeared to perform better on evaluation of residents.

### Strengths and limitations

This study builds on existing body of knowledge on personality traits in relation to job performance and academic performance in medicine, as well as on qualitative research findings on traits of competent teachers in medical education. This is the first study that actually empirically quantified the relations using validated personality and teaching performance measures. In addition, this was the first study to explore this topic across surgical and non-surgical specialties. This resulted in a more nuanced and realistic view on the role of personality traits in teaching practice, as the clinical specialty yields a specific context in which personality traits might have varying costs and benefits [48].

Personality traits were self-reported, which means that the possibility of socially desirable responses should be considered when interpreting the results. Socially desirable reporting is generally higher in situations in which favorable self-presentation is required (e.g. for intended job selections) [52]. As the reporting of personality traits in this study was anonymous and given that this reporting is not part of the documented performance evaluation, we expected little socially desirable reporting. Nonetheless, future research could enhance neutral phrasing of personality items, as neutral phrasing has been shown to decrease the degree of socially desirable answers [53].

Another point of self-reported personality traits is that they might have provided other information about personality traits than observer-reports would have. Indeed, it has been shown that self-reported and observer-reported personality traits each have unique variance [54]. Yet, self- and observer-rated personality traits also showed to have a high degree of construct overlap [54] and self-reported personality traits appeared to provide valid information about the person, predictive for various consequences [17], [55]. Both self- and observer-reported measures deliver valuable information about personality traits, However, both self- and observer-reports of personality (and teaching performance) are indirect measures, and cannot be directly observed (such as blood pressure). The conclusions drawn from our results should be interpreted accordingly.

Up till date, self-reported personality traits in relation to teaching performance are less common in research than other-reported personality traits.[27], [35], [36] Therefore, this study on self-reported personality traits, which also shows associations with teaching performance, makes an original contribution to current knowledge on this topic.

### Explanation of results

The reported positive effects of extraversion are consistent with previous research [27], [35], [36]. This study adds knowledge on the specific teaching skills involved – namely, provision of a motivating learning climate, communication of learning goals, provision of constructive feedback, and adequate evaluation of residents. Possibly, the positive evaluation of extraverted attending physicians reflects residents' appreciation of those attending physicians who are best able to adjust to the demands of modern health care and residency training, which stresses typical extraversion related competencies, such as communication and collaboration [56], [57].

In line with expectations, conscientiousness turned out to be a positive trait for some specific teaching skills, i.e. the creation of a motivating learning climate and communication of learning goals. In general, conscientious people tend to be active learners [58], which may be instrumental or even contagious in terms of teaching residents to be active learners as well. Indeed, residents in this study find that conscientious attending physicians motivate them to study further, keep up with the literature, actively participate in discussions (learning climate) and prioritize learning goals (communication of learning goals).

In addition, we found that attending physicians who reported higher levels of conscientiousness, were perceived as more adequate in evaluating the knowledge and skills of residents, however, this only applied to non-surgical attending physicians. An explanation for this finding could be found in the fact that non-surgical residents find evaluation a more important teaching skill than surgical residents [6]. Therefore, non-surgical residents might appreciate attending physicians who evaluate them conscientiously and thoroughly. In addition, residents' learning process might benefit from conscientious and thorough evaluation of their knowledge and skills regarding patient care cases. Benefits of personality traits (in this case conscientiousness) may depend on the context in which they are expressed (in this case, non-surgical teaching practice) [47], [48]. As such, attending physicians' conscientious evaluation of residents could be seen as a benefit for teaching residents how to recognize and analyze complex clinical cases in non-surgical patient care.

Research on good teaching in medical education highlights that good teachers are personable, approachable and respectful [59]. Indeed, attending physicians who perceived themselves as more agreeable, displayed a more professional attitude towards residents. That is, i.e. these attending physicians were perceived as better listeners, easily approachable and more respectful. Agreeableness however, does not create better teachers in all cases. Unexpectedly, agreeable attending physicians provide less motivating learning climates and evaluate residents less adequately. Agreeableness might hinder attending physicians in evaluating residents and providing a learning climate, as agreeable people tend to avoid confrontations [60]. Indeed, confronting physician colleagues on their way of practicing medicine does not tend to be common practice in the medical profession [61], [62]. Yet, the results of this study suggest that less agreeable behaviors of attending physicians are more favourably evaluated on their adequacy of teaching. Therefore, stimulating the development of the right balance between agreeable and confrontational behavior could be useful in enhancing teaching skills.

We found that surgeons with high levels of openness are less good feedback providers. Corresponding to Netlle's cost-benefit trade-off model [47], [48], high levels of openness might come with certain costs when it comes to giving feedback within the context of surgical teaching practice. Teachers who are open, appear to provide less clear guidelines and structures[63] and possibly, surgical residents prefer a clearer feedback style in learning how to perform surgical operations. In this case, the lack of clear feedback would be a cost of high openness within surgical teaching practice. As we can only speculate about the reason for the context-specific costs of openness, future research could take up this surprising finding.

Contrasting existing evidence [13], [35], our study revealed no relation at all between emotional stability and overall (or domain-specific) teaching performance. Possibly, the lack of an effect can be clarified by a curvilinear relationship between emotional stability and performance, perhaps because both low and high levels of emotional stability might not facilitate performance, while intermediate levels could [15]. That is, a lack of emotional stability could lead to too much strain, while excessive stability — or a lack of anxiety — could lead to lower motivation to invest much energy in training residents. This could mean that attending physicians are served best with intermediate levels of emotional stability. Future research could examine this possibility.

### Implications

In providing adequate training to residents, attending physicians could reflect upon the role of their personality and specialty in relation to their teaching. Teaching teams could ensure quality of teaching by creating heterogeneous teams in terms of personality of attending physicians, as different personality traits have been shown to facilitate different teaching skills. This way, residents can, for example, enjoy the superior professional attitude of their agreeable teachers and discuss learning goals with their conscientious supervisors. Future research could examine the interplay between personality of the resident and personality of the attending physician, as personality of the resident could determine their preference for supervision of specific attending physicians.

As personality traits are broad domains, future research could use extensive personality questionnaires that give more detailed information about the specific personality traits having effect. Then it could become clear, for example, that it is the specific aspect assertiveness of extraverted attending physicians that serves their communication of learning goals. Insights resulting from future research could give direction to development or expansion of training or coaching of (teaching) competences (e.g. assertiveness) of attending physicians.

## Conclusions

This study found that extraverted attending physicians were favorably evaluated on overall and domain-specific teaching performance, signifying the importance of interpersonal and communicative capacities for teaching of attending physicians in residency training. Teaching qualities represent a variety of skills and appear to be served by a variety of personality traits as well. Lastly, what works in one specialty does not seem to work for another specialty. Future research could focus on specific specialties beyond broad categorizations into surgical versus non-surgical specialties, in order to provide more nuanced and detailed inferences.

## Supporting Information

Figure S1 **The associations between mean scores on openness and mean scores on feedback, separately for surgical and non-surgical specialties.**
(TIF)

Figure S2 **The associations between mean scores on conscientiousness and mean scores on evaluation of residents, separately for surgical and non-surgical specialties.**
(TIF)

Figure S3 **The associations between mean scores on openness and mean scores on evaluation of residents, separately for surgical and non-surgical specialties.**
(TIF)
