# Supplementary figures and images for: Personality Traits Affect Teaching Performance of Attending Physicians: Results of a Multi-Center Observational Study
Source: PLoS One. 2014 May 20;9(5):e98107. doi: 10.1371/journal.pone.0098107 (PMC4028262; doi:10.1371/journal.pone.0098107)

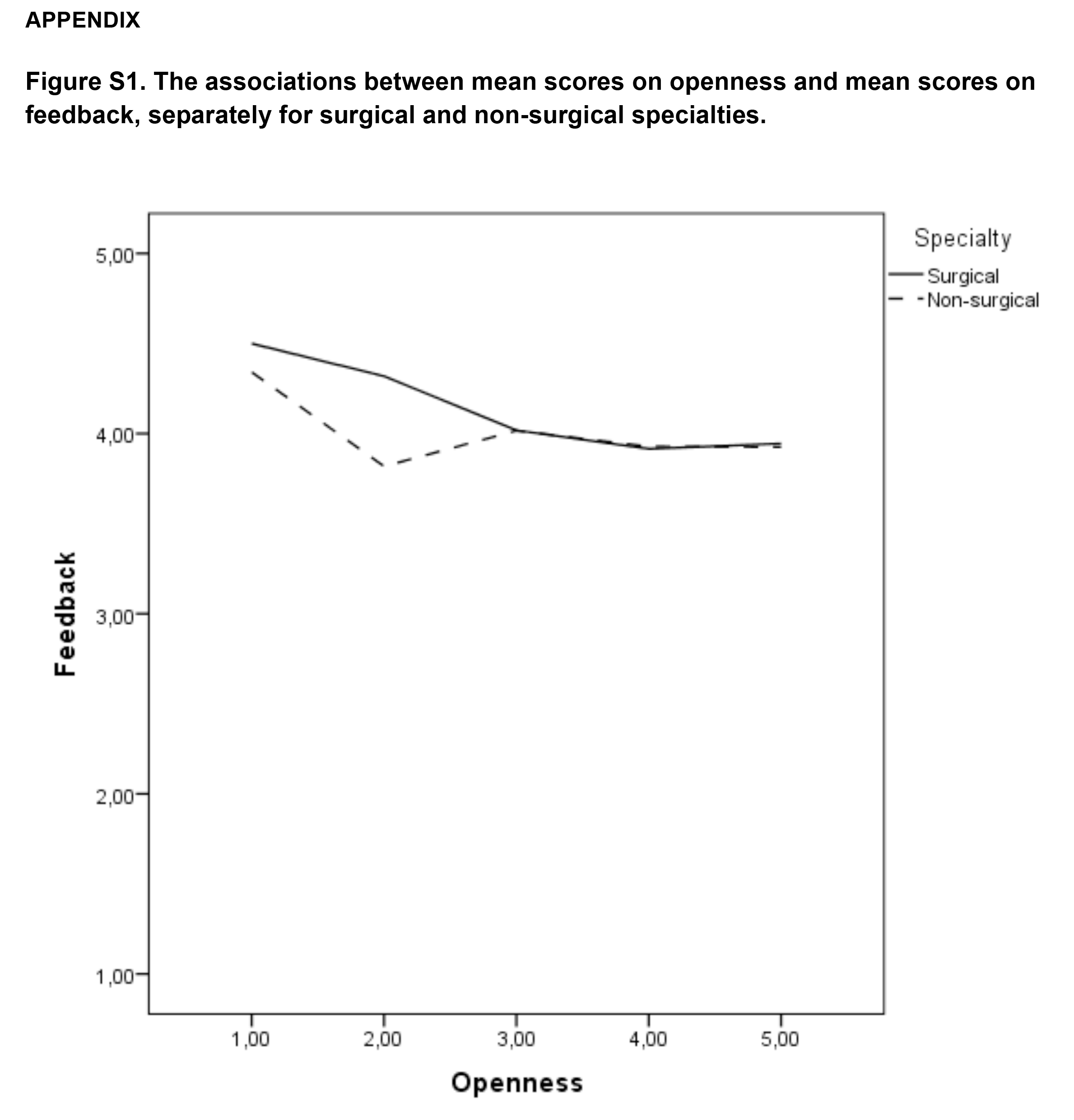

Supplement: Figure S1 — The associations between mean scores on openness and mean scores on feedback, separately for surgical and non-surgical specialties. (TIF) [file pone.0098107.s001.tif]

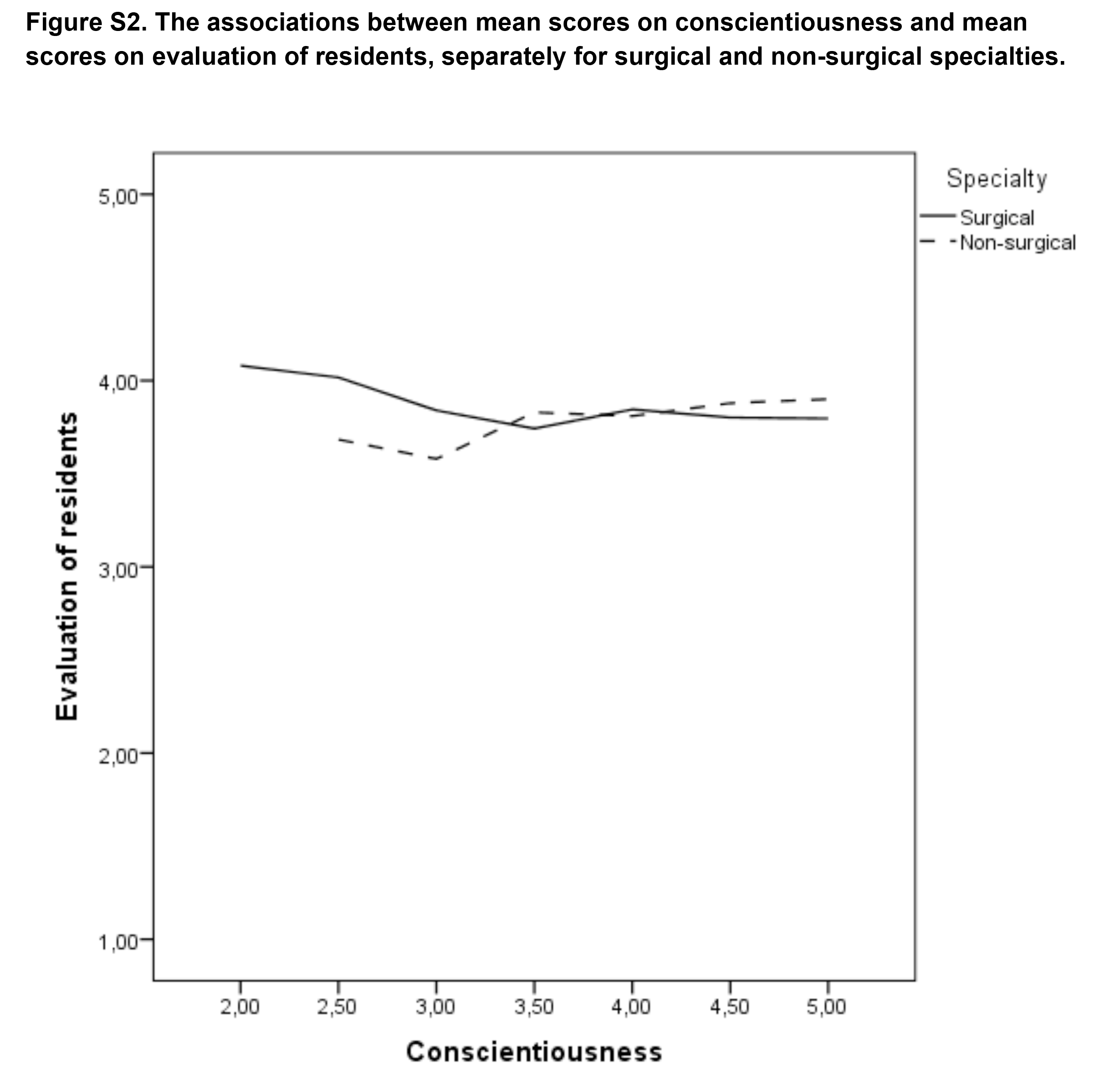

Supplement: Figure S2 — The associations between mean scores on conscientiousness and mean scores on evaluation of residents, separately for surgical and non-surgical specialties. (TIF) [file pone.0098107.s002.tif]

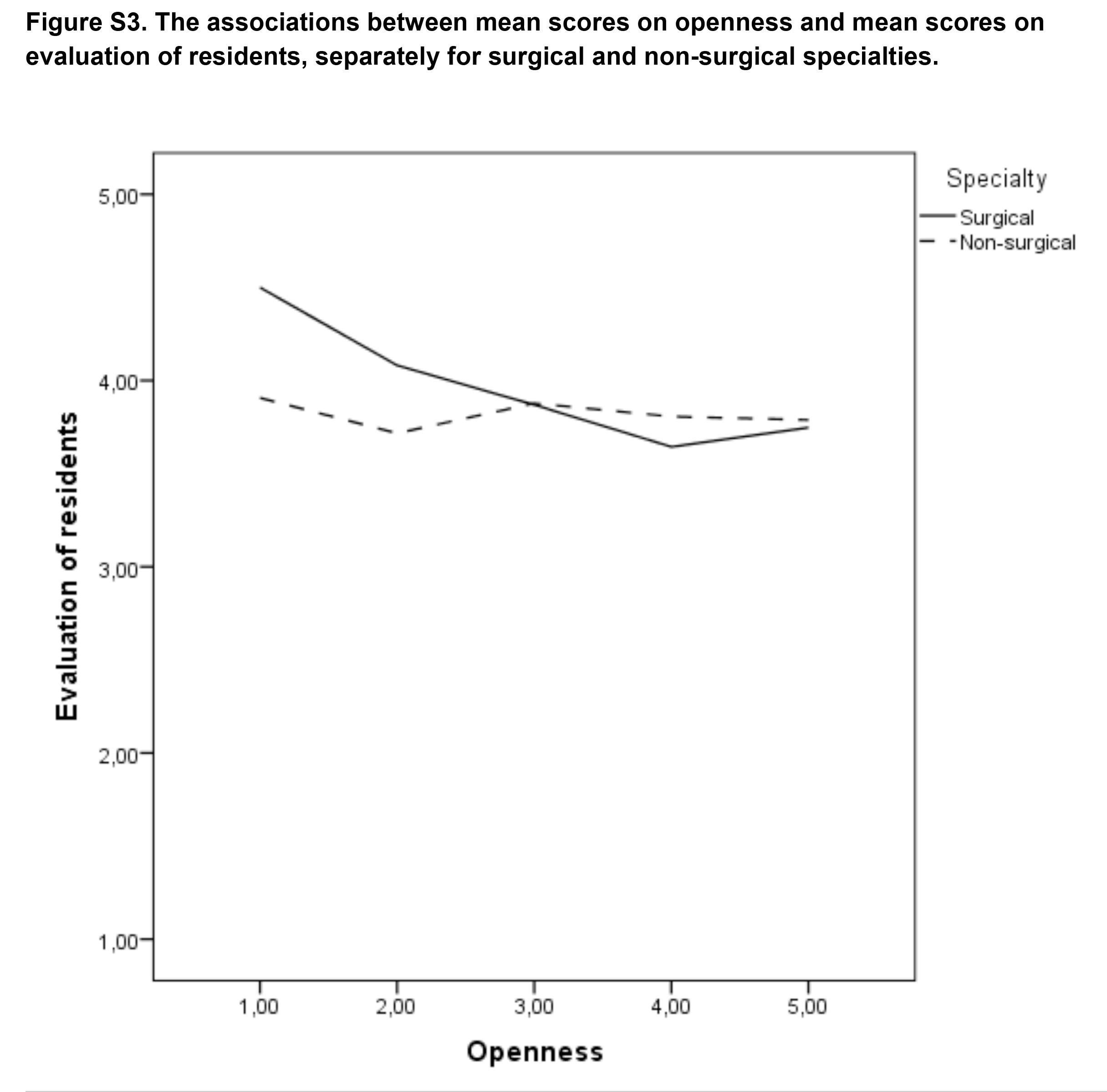

Supplement: Figure S3 — The associations between mean scores on openness and mean scores on evaluation of residents, separately for surgical and non-surgical specialties. (TIF) [file pone.0098107.s003.tif]
